# The Role of Astrocytes in Multiple Sclerosis Progression

**DOI:** 10.3389/fneur.2015.00180

**Published:** 2015-08-18

**Authors:** Jorge Correale, Mauricio F. Farez

**Affiliations:** ^1^Department of Neurology, Institute for Neurological Research Dr. Raúl Carrea, FLENI, Buenos Aires, Argentina

**Keywords:** multiple sclerosis, astrocytes, multiple sclerosis progression, microglia, myelin, axon, glial scar, mitochondria

## Abstract

Multiple sclerosis (MS) is an inflammatory disorder causing central nervous system (CNS) demyelination and axonal injury. Although its etiology remains elusive, several lines of evidence support the concept that autoimmunity plays a major role in disease pathogenesis. The course of MS is highly variable; nevertheless, the majority of patients initially present a relapsing–remitting clinical course. After 10–15 years of disease, this pattern becomes progressive in up to 50% of untreated patients, during which time clinical symptoms slowly cause constant deterioration over a period of many years. In about 15% of MS patients, however, disease progression is relentless from disease onset. Published evidence supports the concept that progressive MS reflects a poorly understood mechanism of insidious axonal degeneration and neuronal loss. Recently, the type of microglial cell and of astrocyte activation and proliferation observed has suggested contribution of resident CNS cells may play a critical role in disease progression. Astrocytes could contribute to this process through several mechanisms: (a) as part of the innate immune system, (b) as a source of cytotoxic factors, (c) inhibiting remyelination and axonal regeneration by forming a glial scar, and (d) contributing to axonal mitochondrial dysfunction. Furthermore, regulatory mechanisms mediated by astrocytes can be affected by aging. Notably, astrocytes might also limit the detrimental effects of pro-inflammatory factors, while providing support and protection for oligodendrocytes and neurons. Because of the dichotomy observed in astrocytic effects, the design of therapeutic strategies targeting astrocytes becomes a challenging endeavor. Better knowledge of molecular and functional properties of astrocytes, therefore, should promote understanding of their specific role in MS pathophysiology, and consequently lead to development of novel and more successful therapeutic approaches.

## Introduction

Multiple sclerosis (MS) is an inflammatory disorder causing central nervous system (CNS) demyelination and axonal injury. Although its etiology remains elusive, several lines of evidence support the concept that autoimmunity plays a major role in disease pathogenesis (1).

The course of MS is highly variable; nevertheless, most patients initially present a relapsing–remitting clinical course [relapsing–remitting MS (RRMS)]. After 10–15 years of disease, this pattern becomes progressive in up to 50% of untreated patients, during which time clinical symptoms slowly cause constant deterioration over a period of many years [secondary progressive MS (SPMS)]. In about 15% of MS patients, however, disease progression is relentless from disease onset [primary progressive MS (PPMS)] (2).

In recent decades, better understanding of RRMS disease mechanisms has led to development of different disease-modifying therapies, reducing both severity and frequency of new relapses, by modulating or suppressing the immune system (3). By contrast, therapeutic options available for progressive MS are comparatively disappointing, and remain challenging. One possible reason behind this is a lack of understanding of the pathogenic mechanisms driving progressive MS.

The conventional view explaining the sequence of MS events is one in which systemic activation of myelin reactive cells from the periphery migrate into the CNS, leading to inflammation and development of focal demyelinating lesions, which constitute the main pathological substrate for relapses. The progressive phase of MS reflects a poorly understood and insidious form of axonal degeneration with neuronal loss, independent of relapses. Pathological studies have shown that axonal degeneration occurs diffusely throughout normal appearing white matter (4). Although this neurodegenerative component is associated with inflammation (5), there is growing awareness that T cell-mediated inflammatory mechanisms alone, cannot explain the degenerative process. Recently, a number of observations have challenged the concept of an autoimmune attack against myelin mediated only by adaptive immune response to self antigens, as the complete and full explanation behind the disease, particularly during its progressive phases. For example, pathology studies of early lesions show oligodendrocyte and myelin loss, in the absence of T or B cell infiltrates (6). Likewise, large areas of myelin loss are seen in cerebral cortex and deep gray matter nuclei with a paucity of infiltrating immune cells (7–9). Activation and proliferation of microglia and astrocytes observed within demyelinating lesions suggest that innate immune response contribution by resident CNS cells might play a critical role in both oligodendrocyte injury and axonal degeneration (10). Indeed, glial cells and astrocytes, in particular, were found to be highly abnormal, early in the study of MS lesions (11). Large and bizarre astrocytes containing multiple and sometimes fragmented nuclei or engulfing other cells were found in early active lesions (12), and considered by investigators during the late nineteenth and early twentieth century, to be the major cell type targeted in MS (13). However, later identification of oligodendrocytes as the myelinating cell of the CNS, as well as of their depletion from MS lesions, caused the role of astrocytes in MS pathogenesis to be largely ignored after about 1930. Nevertheless, most neuropathologists continue to report astrocyte appearance in MS lesions, and consider it an important indicator of lesional activity and age (14).

This review summarizes current studies on the role of astrocytes in disease progression, and discusses data on some of the mechanisms through which these cells may play a key role in MS pathogenesis.

## Overview of Astrocytes

Astrocytes are the most abundant and heterogeneous type of glial cell (15). Two main subtypes exist: fibrous and protoplasmic, based on cell morphology and anatomical location. Fibrous astrocytes of the white matter have small cell bodies, and their processes align with myelinated fibers, giving them an elongated morphology (16). Protoplasmic astrocytes have more primary processes, as well as a higher degree of branching compared to fibrous astrocytes, and are located in the gray matter (17). Additionally, other morphologically distinct and more regional populations of astrocytes have been described, such as Müller cells in the retina, Bergmann glia and velate astrocytes in the cerebellum, radial astrocytes in the spinal cord, among others (18). Consequently, astrocytes can no longer be considered as a homogeneous group of cells. Their morphological diversity, specific density, as well as proliferation rate will be determined by interactions with the microenvironment, particularly during development, reflecting important molecular and functional differences between astrocyte types (19, 20).

Astrocytes have at least two different origins: (1) directly from radial glial cells located in the ventricular zone and (2) from a proliferative and migratory population located in the subventricular zone (SVZ) (21–24). New astrocytes may arise either from the proliferation of mature astrocytes or from differentiation of progenitors. Notably, there is little evidence that mature astrocytes divide in the uninjured brain (19). By contrast, very active proliferation is associated with scar formation following injury (see below).

Astrocyte development is regulated by different molecules and through different intracellular pathways including the IL6/LIF family of cytokines, the TGF-β growth factor family, fibroblast growth factor (FGF), and Notch and Notch ligand pairs (25). Additionally, epigenetic factors also influence astrocyte development. A number of astrocyte genes are methylated early in development, including *GFAP, Aldolase C*, and *Kir4.1*, a process that serves to repress astrocyte specific gene transcription (26). By contrast, demethylation occurring during early astrocyte development allows LIF to upregulate genes, through binding to transcription factors present downstream to astrocyte gene promoters in the signaling pathways (26, 27).

Expression of glial fibrillary acidic protein (GFAP) has become the prototypic marker for identifying astrocytes within the CNS; however, expression patterns differ across anatomical regions (28). Moreover, other CNS-resident cells, such as NG2 and pericytes, have also been to shown to be GFAP^+^ (9). Several other antibodies against intermediate filament proteins, including cytoplasmic or membrane protein markers, such as vimentin, nestin, S100 calcium-binding protein β (S100β), glutamine synthetase (GS), or glutamate/aspartate transporter (GLAST), are also commonly used to label normal and reactive astrocytes (28). A significant drawback of current immunohistochemistry techniques is that no reliable markers exist to identify different astrocyte subtypes, making it hard to establish whether any given behavior observed corresponds to astrocytes in general, or is characteristic of a particular subtype only.

Astrocytes contact blood vessels and are linked to each other via gap junctions, and to oligodendrocytes via heterotypic gap junctions. Adjacent astrocytes present homomeric gap junctions at the cytoplasmic level, formed by connexin (Cx) 43 and Cx 30, through which molecules, such as K^+^ and glutamate, are dissipated, and intercellular Ca^++^ waves propagate (29). In addition, astrocytes support several activities essential for neuronal function, including (1) an active role in both formation and pruning of synapses (30); (2) regulation of extracellular concentrations of ions and neurotransmitters (29, 31); (3) synthesis of metabolic substrates for neurons, such as glycogen, sterols, and lipoproteins (23, 32); (4) formation and maintenance of blood–brain barrier (BBB) integrity, thus protecting the brain from influx of toxic substances and ions, as well as maintaining extracellular space volume (33); and (5) removal of neurotransmitters released by active neurons, such as glutamate (34). Central questions remaining include whether astrocytes in general carry out all these functions, and if not, what relevance differences in subpopulations may play in human disease.

## Astrocytes in Multiple Sclerosis

### Contribution of astrocytes to MS lesion development

Around acute inflammatory lesions, astrocyte reactivity is widespread. A gradient of response is observed, ranging from modestly swollen process-bearing cells in normal adjacent white matter, to hypertrophic astrocytes in the center of a lesion (14, 35). As lesions age not only persist hypertrophic astrocytes but also begin to develop bundles of glial filaments, GFAP immunoreactivity increases, and edema decreases. Relapsing disease activity is associated with recurrent inflammatory activity, astroglial reactivity (particularly along lesion borders), and recent astrocyte mitotic activity (14, 35). Studies in experimental autoimmune encephalomyelitis (EAE) have shown that activation of astrocytes, and loss of their end-feet around small blood vessels represent early events in lesion development, linked to loss of BBB function, subsequent CNS inflammation, as well as perivascular edema (14). It is well recognized that factors produced by astrocytes are required for establishment and maintenance of endothelial cells forming the BBB. For example, astrocyte activation by macrophage produced IL-1, leads to induction of hypoxia inducible factor-1 (HIF-1), and its target, vascular endothelial growth factor A (VEGF-A) in astrocytes, which acting on endothelial cells induces down-regulation or loss of tight proteins claudin-5 (CLN5) and occludin, determining a focal loss of BBB function in injured tissue, a process mediated by eNOS (36, 37). Inactivation of VEGF-A expression, or systemic selective inhibition of eNOS reduces BBB breakdown, decreasing lymphocyte infiltration and tissue damage, protecting against neurological deficit in EAE (38). Besides tight junctions on endothelial cells, astrocyte end-feet forming glia provide an additional barrier against autoreactive cell activity in the CNS. Furthermore, imbalance between upregulation of matrix metalloproteinases (MMPs) in both astrocytes and macrophages, compared to stable expression or reduction of parenchymal basal membrane components aid in encephalitogenic cell dispersion into the CNS (39). However, it should be noted that remodeling of the extracellular matrix (ECM) can be both deleterious and beneficial, depending on the situation and on the type of MMP involved (Table 1) (14, 20, 39).

**Table 1:** **The dual role of astrocytes in the pathophysiology of multiple sclerosis**.

Deleterious roles	Protective/remyelinating roles
Recruitment of T cells, macrophages and microglia cells to CNS lesion	Modulation of BBB integrity: secretion of TIMPs
Chemokine production	
Modulation of adhesion molecules (VCAM-1 and ICAM-1)	
Modulation of BBB integrity (VEGF-A and HIF-1)	
Secretion of MMPs	

Activation of immune response	Termination of the immune response
Secretion of pro-inflammatory cytokines (IL-1β, IL-6, IL-12, IL-17, IL-23; TNF-α)	Induction of apoptosis (Gal 9-Tim-3 interaction)
IL-15-driven cytotoxic activity of CD8^+^ T cells	Support differentiation of Treg cells (TGF-β, IL-10, IL-27)
Production of BAFF contributing to B-cell dependent autoimmunity	Secretion of anti-inflammatory cytokines (IL-10, TGF-β, IL-27)
	Microglia inhibition (Gal-1)

Inhibition of axonal regeneration	Viability of neurons: secretion of NT-3, BDNF, and CNTF
Secretion of CSPGs	
NOGO-NgR-TROY-LINGO interactions	
Secretion of ephrins	

Secretion of cytotoxic factors: NO, ROS, purinergic metabolites	Prevention of excitotoxicity by glutamate uptake

Inhibition of remyelination	Promotion of remyelination
Regulation of NG2/OPC migration (glial scar)^a^	Glial scar formation^a^
Secretion of FGF-2 prevents OPC maturation	Modulation of NG2/OPCs survival, proliferation and differentiation into Oligodendrocytes (IL-6, IL-11, LIF, IGF-1, FGF-2)
Production of semaphorin 3A produces OPC repulsion	Production of semaphorin 3F producing OPC attraction
Notch/Jagged 1 interaction arrested OPC in an immature state	Myelin breakdown clearance (phagocytosis)^b^

Secretion of LacCer	
Induces activation of microglia (GM-CSF)	
Induces chemotaxis of monocytes (chemokine CCL2)	

TGF-β production induces a SASP phenotype	

Release of HMGB1 (secretion of MMP-9, cyclo-oxigenase2 and chemokines	

Antigen presenting cell function (?)^b^	

*BAFF, B-cell activating factor; BBB, blood–brain barrier; BDNF, brain-derived neurotrophic factor; CNS, central nervous system; CNTF, ciliary neurotrophic factor; CSPGs, chondroitin sulfate proteoglycans; FGF, fibroblast growth factor; Gal, galectin: GM-CSF, granulocyte-macrophage colony-stimulating factor; HIF-1, hypoxia inducible factor-1; HMGB1, high-mobility group box-1; ICAM-1, intercellular adhesion molecule-1; IGF-1, insulin growth factor; LacCer, lactosyceramide; LIF, leukemia inhibitory factor; MMPs, metalloproteinases; NG2, neuron glial antigen; NgR, NOGO receptor; NO, nitric oxide; NT-3, neurotrophin-3; OPC, oligodendrocyte precursor cells; ROS, reactive oxygen species; SASP, senescence-associated secretory phenotype; Tim, T cell immunoglobulin domain; TIMPs, tissue inhibitors; Treg, regulatory T cell; VCAM-1, vascular adhesion molecule-1; VEGF-A, vascular endothelial growth factor A*.

*^a^Glial scar can impact both beneficially and detrimentally on surrounding neuronal and non-neuronal cells*.

*^b^Antigen presenting cell function and phagocytosis by astrocytes remains unclear under physiological conditions*.

It is important to point out the dual role of astrocytes, not only aiding in axonal degeneration and demyelination but also creating a permissive environment promoting remyelination (Table 1). The particular impact of astrocytes on pathogenesis and repair of an inflammatory process, therefore, will be dependent on a number of factors, including timing after injury, type of lesion and surrounding microenvironment, as well as interaction with other cell types and factors influencing their activation (39).

### Astrocytes and the innate immune system

Innate immunity is the initial non-specific response to foreign pathogens. The system includes cellular barriers, such as the BBB and diverse immune cells of myeloid origin, including DCs, macrophages, monocytes, NK cells, NKT cells, mast cells, granulocytes and γδ T cells in the periphery and microglia cells in the CNS. Innate immunity also includes non-myeloid cells, such as astrocytes (9). Cellular innate immune responses to diverse stimuli are accomplished through an array of pattern recognition receptors (PRRs) that bind to diverse pathogen-associated molecular patterns (PAMPs) (40). Notably, PRRs also recognize self-molecules released after cell damage or death. These molecules, known as danger-associated molecular patterns, include diverse ligands, such as heat-shock proteins, double stranded DNA, and purinergic metabolites (9, 41). Responses to endogenous host molecules may trigger inflammatory reactions, and therefore play an important role in autoimmunity.

Astrocytes express diverse PRRs, and can mediate innate immune responses through several mechanisms (10, 42). First, astrocytes directly affect cell entry to the CNS, via the BBB, by regulating expression of adhesion molecules, particularly vascular adhesion molecule-1 (VCAM-1) and intercellular adhesion-molecule-1 (ICAM-1) that bind to lymphocyte receptors, namely, very late antigen-4 (VLA4) and lymphocyte function-associated antigen-1 (LFA-1), respectively (43, 44). In addition, release of IL-6, IL-1β, TNF-α, and TGF-β by astrocytes can control passage of immune cells through the BBB, by acting on endothelial cells and tight junctions (33, 45, 46).

Second, astrocytes secrete different chemokines, such as CCL-2 (MCP-1), CCL5 (RANTES), IP-10 (CXCL10), CXCL12 (SDF-1), and IL-8 (CXCL8), which attract both peripheral immune cells (e.g., T cells, monocytes, and DCs), as well as resident CNS cells (microglia) to lesion sites (47). This could represent the primary mechanism through which astrocytes perpetuate immune-mediated demyelination and neurodegeneration. *In vitro* studies confirm that human astrocytes secrete IP-10, CCL-2, and CXCL12 in response to inflammatory cytokines IL-1β, TNF-α and IFN-γ, suggesting astrocyte-induced immunopathology may be a consequence of activation by infiltrating T cells (48–50).

Third, astrocytes may affect both the number and the phenotype of T cells in the CNS. Cytokines secreted by astrocytes have the potential of committing T cells to a pro-inflammatory phenotype (Th1 and Th17) or to a regulatory phenotype (Treg, Tr1). Under inflammatory conditions astrocytes express all subunits of IL-12/IL-23, as well as CD24, favoring the development of Th17 and Th1 cells in the CNS during EAE, thereby affecting its severity (51, 52). Additionally, IL-9 receptor complex is constitutively expressed in astrocytes, T cell-derived IL-9 induces astrocytes to produce CCL20, which in turn induces Th17 cell migration *in vitro* (53). Treatment with anti-IL-9 neutralizing antibodies attenuates EAE, decreasing the number of infiltrating Th17 cells, and reducing CCL-20 expression in astrocytes (53). Furthermore, astrocyte-driven IL-15 production, which has been observed in MS lesions, has been shown to have an important role in encephalitogenic activity of CD8^+^ T cells (54). By contrast, astrocytes can also terminate T cell responses, either by induction of apoptosis of infiltrating cells through FAS-L, which is highly expressed on astrocyte end-feet (55), or through interaction of galectin-9 and its ligand Tim-3, present in Th1 and CD8^+^ cytotoxic T cells (56).

Fourth, B-cell-activating factor (BAFF), critical for both B cell development and survival, as well as for the production of immunoglobulins, is constitutively expressed by astrocytes in normal CNS. BAFF expression in astrocytes is upregulated in MS lesions and in EAE affected mice, suggesting astrocytes may contribute to drive B-cell-dependent autoimmunity (57).

Fifth, astrocytes modulate microglial and macrophages activity through two different pathways: (a) inducing their recruitment toward lesion sites by producing chemotactic signals (CXCL-10-CXCR3) (58) and (b) by secreting GM-CSF, M-CSF, or TGF-β, which can regulate Class II expression, and even microglial phagocytosis (59).

Finally, an important function of innate immune cells is to act as antigen presenting cells (APCs). However, although astrocytes express major histocompatibility complex (MHC) class I and class II molecules *in vitro* capable of presenting myelin antigens, their ability to also express co-stimulatory molecules including CD40, CD80, and CD86 challenges this function, making their final effect unclear (60, 61). Nor is it clear to what degree astrocytes can perform phagocytosis, or process and present antigens, particularly under physiological conditions *in vivo* (62).

Recent investigations have demonstrated that in chronic phases of EAE, astrocyte depletion ameliorates disease severity. This deleterious effect of astrocytes on EAE is mediated by preferential expression of 4-galactosyltransferase 5 and 6 (B4GALT5 and B4GALT6) (63). Notably, in human MS lesions, B4GALT6 is expressed by reactive astrocytes. These enzymes synthesize the signaling molecule lactosylceramide (LacCer), the expression of which is significantly increased in the CNS during progressive phases of EAE. Furthermore, intraperitoneal administration of LacCer exacerbates existing signs of EAE. LacCer promotes astrocyte activation in an autocrine manner, via the NF-κB and IRF-1 pathways (63, 64), leading to inducing GM-CSF and CCL2 genes, consequently activating microglia and causing infiltration of monocytes from blood, respectively. Remarkably, inhibition or knockout of *B4galt6* in mice suppresses disease progression, local CNS innate immunity, and neurodegeneration in EAE, and interferes with human astrocyte activation *in vitro* (63).

### Astrocytes as a source of cytotoxic factors

In most areas of myelin breakdown, it has been documented that activated astrocytes secrete compounds with toxic effects on neurons, axons, and oligodendrocytes/myelin, including reactive oxygen and nitrogen species, glutamate, and ATP (14). In rodents, astrocytes stimulated with IFN-γ, IL-17, or LPS induce nitric oxide synthase (iNOS) (65, 66). Likewise, IL-1β as well as combined treatment with TGF-β plus IFN-γ increases percentage of astrocyte secreted nitric oxide (NO), which is among the most prominent damage-inducing molecules in neurodegeneration (67, 68). Moreover, *in situ* hybridization and immunohistochemistry of astrocytes in MS, as well as in EAE lesions, demonstrates extensive iNOS reactivity and positive nitrotyrosine presence (69, 70). Furthermore, recent studies suggest a strong relationship between excessive calcium influx mediated by glutamate receptor stimulation (see below), and increased NO synthase activity, as well as amplified formation of reactive oxygen species (ROS), providing a link between excitotoxic insult and NO-mediated damage. Simultaneously, excitotoxicity is further increased by NO, which stimulates glutamate release from astrocytes (71). Remarkably, the predominant contribution of NO to excitotoxicity depends on increased superoxide ion ${\text{O}}_{2}^{-}$ production, which reacts with NO forming peroxinitrite (ONOO^−^), resulting in neuronal necrosis or apoptosis, depending on its concentration (72). Furthermore, ONOO^−^ inactivates glutamate transporters in astrocytes, directly damaging myelin, oligodendrocytes, and axons (73).

Decreased uptake of glutamate by astrocyte transporters could also contribute to pathologically elevated levels of extracellular glutamate, which are directly toxic to oligodendrocytes, axons, and neurons (74). In mice, TNF-α from cortical astrocytes down-regulates expression of glutamate transporters in astrocytes, thus limiting glutamate uptake (75). Furthermore, knock down of glutamate transporters: GLAS and GLT-1 using antisense oligonucleotide causes neurotoxicity in mice (76). Excitotoxicity is caused mainly by sustained activation of glutamate receptors and massive subsequent influx of Ca^++^ into viable neurons. Calcium, which is the primary signaling agent involved in excitotoxicity injury, enters cells through various mechanisms, but the most important is entrance through ion channels coupled to NMDA receptors and AMPA/kainate glutamate receptors (77, 78). Calcium overload determined by glutamate receptor activation, in turn, activates several Ca^++^-dependent enzymes associated with neurodegeneration and cell death by causing membrane breakdown, cytoskeleton alteration, and NO-derived free radical formation. Moreover, intracellular calcium increase results in changes in microtubules and neurofilament phosphorylation, which ultimately leads to axon cytoskeleton breakdown (79, 80). Recent studies have shown glutamate can also be toxic to white matter oligodendrocytes and myelin, via mechanisms triggered by AMPA/kainate receptors (81). Indeed, treatment with glutamate receptor antagonists protects oligodendrocytes from damage, ameliorating EAE (82). Thus, proper function of glutamate uptake in astrocytes is critical to preclude brain cell damage, and strict regulation of extracellular glutamate levels appears to be a very prominent therapeutic strategy preventing neurodegeneration in MS.

Extracellular purine/pyrimidine metabolites are also exogenous signals playing important destructive/protective roles in neuron to glia, or glia to glia communication within normal or injured brain tissue. They activate membrane-bound ionotropic or metabotropic P2 receptors. Astrocytes express various types of metabotropic P2Y metabotropic, and ionotropic P2X purinoreceptors. Studies in MS lesions have shown preferential expression of P2X7 receptor on astrocytes (83). Although expression is low in resting human fetal astrocytes, P2X7 is upregulated in response to IL-1β *in vitro* and in reactive astrocytes around MS lesions, a putative IL-1β rich environment (84). Functionally, upregulation of P2X7 results in increased responsiveness to ATP, formation of membrane pores, and increased influx of Ca^++^ (85). Furthermore, purinergic signaling through P2X7 receptors stimulates IL-1β-induced upregulation of NO synthase (84). Thus, activation of the P2X7 receptor in EAE can trigger toxic effects on oligodendrocytes, axons, and neurons through different mechanisms, producing *in vivo* lesions reminiscent of MS plaques, displaying oligodendrocyte death, demyelination, and axonal damage.

### Astrocytes inhibit remyelination and axon regeneration by forming a glial scar

Astrocytes respond to injuries through a process commonly referred to as reactive astrogliosis, which involves changes in cell morphology and molecular expression. It is important to remember that although some aspects of glial reactivity are likely to be protective, others may contribute to disease progression. Establishing the molecular basis of such differences may therefore help identify novel therapeutic strategies. Although the best known aspect of reactive astrogliosis is scar formation the concept of reactive astrogliosis is still incomplete, we are only just starting to understand its molecular and cellular characteristics, as well as its multifaceted functions in disease pathogenesis and in CNS recovery from injury. The scar is composed primarily of astrocytes, however, in severe lesions, interaction with other cell types including oligodendrocyte progenitor cells (OPCs) and fibromeningeal cells also occurs (86, 87). Several specific molecular and morphological features have been observed in astrocytes during reactive astrogliosis in both human pathology and animal models (88, 89), of which upregulation of GFAP, vimentin, nestin, and the less investigated synemin are hallmarks. A number of other molecules, such as TGF-α, ciliary neurotrophic factor (CNTF), LIF, and oncostatin M, trigger astrocyte activation in the rodent brain (90). Interestingly, levels of IL-6, LIF, and oncostatin M mRNA, all ligands in the gp130/activator of transcription 3 (STAT3) signaling pathway, are elevated prior phosphorylation and nuclear transcription of STAT 3, both in astrocytes and during astroglyosis induction (91). Nevertheless, it is also conceivable that at least some of these molecules exert effects on astrocytes through other cell types, such as microglia, neurons, or endothelial cells. By contrast, signaling mediated by β1-integrin has the opposite effect on astrocyte activation and is required to promote development of a mature non-reactive astrocyte (92). Other mechanisms may also contribute to astrogliosis. It has been shown that inositol 1,4,5-triphophate (IP3)-dependent Ca^2+^ signaling and the downstream functions of N-cadherin in astrocytes are required for normal reactive astrogliosis (93). Likewise, epidermal growth factor receptor (EGFR) has been implicated in astrocyte transition from a non-reactive to a reactive state (94). Moreover, astrocytes react to endogenous or exogenous ATP with hypertrophy, swelling cell body and main processes, and generating proliferation, ultimately resulting in an astrogliosis phenotype, which subsequently forms a glial scar (83). ATP *per se* can trigger these biological effects through activation of P2 receptors (P2R), or through its metabolites ADP, activating some P2R and adenosine through P1R activation (95). Another factor that might contribute to astrocyte ability to react to injury is cell polarity and migration: astrocytes depleted of RhoGTPase Cdc42, a key regulator of polarization, impaired recruitment to lesions despite GFAP upregulation and hypertrophic response (96). It is important to note that reactive astrogliosis is, at least partially, disease specific. For example, reactive astrocytes profoundly affect post-ischemic stages by secreting VEGF, which in turn stimulates formation of new blood vessels and synaptogenesis (97, 98), this beneficial effect contrasts sharply with induction of BBB breakdown and lymphocyte infiltration observed in autoimmune CNS inflammation, which worsen disease (38).

Several experimental approaches have been used to either eliminate reactive astrocytes, or prevent them from becoming fully reactive. Thus, infiltration of CD11b^+^ microglia/monocytes in a retinal detachment model was blocked in GFAP^−/−^ Vim^−/−^ mice, suggesting activated glial cells are critical for recruitment of microglia/monocytes to injured areas (99). Similarly, GFAP or nestin promoter ablation of STAT3 in astrocytes attenuated upregulation of GFAP, reduced astrocytes hypertrophy, limited astrocyte migration, and led to more widespread infiltration of CD11b^+^ inflammatory cells, associated with larger lesions and more prominent impairment (100, 101). Conversely, mice with nestin promoter-driven ablation of SOCS3, which inhibits STAT3 signaling, showed increased astrocyte migration, and enhanced contraction of lesions as well as improvement of functional recovery after spinal cord injury (101). Also, ATP released from damaged cells after injury, acting via P2Y receptors enhanced the proliferative effects of FGF2, whereas P2X receptor stimulation inhibited the ability of FGF2 to stimulate DNA synthesis in astrocyte cultures (102). These variable effects of ATP and of other purinergic ligands are mediated by phosphorylation of different STAT3 residues (103). Therefore, pharmacological antagonists of P2X/P2Y receptors might ameliorate long-lasting consequences of different CNS injuries. Overall, these results point to an important role of STAT3 signaling in CNS injury, which may limit development of a potential toxic environment by the rest of the CNS (100, 101), although this might also restrict regenerative responses at a later stage [Ref. (104); see below]. Consequently, there is urgent need for better understanding of the molecular pathways regulating distinct aspects of reactive astrogliosis, in order to allow selective blockade of molecules inhibiting axonal outgrowth, but still permit reactive astrocytes to form a protective scar.

Glial scars are evident in tissue from MS patients and mice with EAE and surround areas of demyelination (105). The purpose of scar formation would appear to be isolation of damaged CNS areas, to prevent spread of tissue destruction. However, glial scar rigidity results in inhibition of both remyelination and axonal regeneration, both negative effects mediated through different mechanisms. First, astrocytes may be detrimental for remyelination by over secreting FGF-2, which in turn promotes OPC proliferation and survival, but prevents maturation (106). Another molecule that appears to play an important role in preventing OPC maturation is the glycosaminoglycan (GAG) hyaluronan, which is found throughout the ECM and in CNS white matter (107). Hyaluronan is produced by astrocytes, and interacts with CD44, a receptor present on OPCs, astrocytes, and T cells in both MS and EAE CNS tissue (19, 108). Oligodendrocytes that co-localize with hyaluronan express an immature phenotype, and treatment of OPCs with hyaluronan *in vitro* prevents maturation (109).

Second, astrocytes release inhibitory ECM molecules known as chondroitin sulfate proteoglycans (CSPGs) in injured areas (110). CSPGs are a family of molecules characterized by a protein core to which highly sulfated GAG chains are attached. Three types of CSPGs are preferentially localized to astrocytes *in vivo*: neurocan, brevican, and NG2. Neurocan (secreted) and brevican (cell bound) are the major proteoglycans produced by astrocytes *in vitro* and both have been shown to inhibit axon growth, following CNS damage (104). There is clear evidence that CSPGs are produced in excess by astrocytes when they become reactive and that inhibitory activity of CSPGs depends on the GAG component, as removal of GAG chains from the protein core eliminates inhibition (104, 111). After injury, CSPGs expression is rapidly upregulated by reactive astrocytes, forming an inhibitory gradient that is highest at the center of lesions and diminishes gradually toward the periphery (112). Meanwhile, NG2 is most often regarded as a marker of OPCs in adult CNS tissue. Along the borders of glial scars, NG2^+^ cells are found in great numbers. While many of these cells are regarded as OPCs, evidence indicates that NG2^+^ cells are also able to become astrocytes *in vivo* (113). Therefore, astrocyte-derived NG2 cells may provide inhibitory signals, suppressing axon regeneration. *In vitro* studies have demonstrated that NG2 inhibits axonal growth, and that this inhibition can be overcome by anti-NG2 antibody treatment (114). CSPG-mediated inhibition could severely affect both cytoskeleton and membrane components of growth cone architecture. In addition, many signaling pathways that mediate inhibition, such as those involving the GTPase RhoA, share similarities with those triggered by myelin-associated inhibitors (MAIs) (see below).

Aside from CSPGs, there are other less studied inhibitory molecules expressed by astrocytes that suppress axonal growth. Ephrins (EPH) and their receptors, for example, are secreted by normal astrocytes and increased in MS lesions (115). Evidence indicates that astrocyte-derived ephrins create a basal lamina around areas of injury, contributing to scar formation. Additionally, ephrins induce collapse of the axonal growth cone through activation of axon-bound EPH tyrosine-receptor kinase (116).

Finally, MAIs, such as Nogo-A, myelin-associated glycoprotein, and oligodendrocyte myelin glycoprotein, can also inhibit axonal growth (112). These three proteins share two common neuronal receptors NgR1, together with its co-receptors (p75, TROY, and LINGO-1), and the recently described paired immunoglobulin receptor-B (PirB) (117, 118). In addition, new ligands binding to the NOGO receptor complex have been reported: glioma-inactivated gene product (LGI), B lymphocyte stimulator (BLyS), and FGF (119–121). Moreover, new receptors for MAIs have been recently described, such as NgR1 isoform, NgR2, and NgR3 (122). CNS regeneration inhibitors target the actin cytoskeleton thus regulating dendritic spine maturation, as well as long-term synaptic stability and plasticity. Although most evidence shows that many CNS inhibitors and their receptors are present or near synapses, astrocytes express p75, TROY, and BLyS, therefore, interaction with these ligands suggests astrocytes may also inhibit remyelination and axonal regeneration through these pathways (123).

### Astrocytes contribute to axon mitochondrial dysfunction

There is emerging evidence that mitochondrial dysfunction actively contributes to neurodegeneration and axonal damage. Mitochondria are also key for ATP production and calcium signaling regulation (71).

Astrocytes may reduce mitochondrial energy metabolism in axons through different mechanisms. One related to increase NO production. Nitric oxide synthase (NOS2) expression is increased in both active focal lesions and normal appearing white matter (124). Interestingly, immunostaining shows NOS2^+^ cells are predominantly astrocytes. A loss of astrocytic β_2_ adrenergic receptors might explain increased NOS2 expression (125). Indeed, noradrenergic stimulation leads to increased cAMP levels and consequently inhibits NOS2 expression in astrocytes. Elevated levels of NO can compete with oxygen for binding on complex IV of the mitochondrial respiratory chain, reducing electron flow and subsequent ATP synthesis (126); a second mechanism is excitoxicity triggered by increased glutamate levels (see above), and intracellular calcium overload. Increased Ca^++^ influx into axons mediated by overstimulation of glutamate receptors may damage mitochondria by promoting Ca^++^ entry into the matrix, leading to inhibition of respiratory chain complex I, and release of cytochrome *c* into the cytosol (77, 78, 127). Furthermore, excess of intra-axonal Ca^++^ may stimulate a variety of Ca^++^-dependent catabolic enzyme systems, including proteases, phospholipases, and calpains, ultimately leading to progressive cytoskeletal degeneration within axons (128). These observations have been confirmed not only in animal models but also in post-mortem studies of MS patients. A third possible mechanism is impaired glycogenolysis and lactate formation secondary to β_2_ adrenergic receptor deficiency in astrocytes, leading to decreased axonal mitochondrial metabolism and reduced *N*-acetyl aspartate (NAA) synthesis, as well as impaired GS activity (129–131). Reduced NAA may alter myelin membrane turnover, leading to myelin loss. Damage of the myelin sheath may contribute to axonal degeneration by reducing trophic support and impairing axonal transport. Overall, evidence is accumulating that defective axonal energy metabolism may cause diffuse axon degeneration observed in MS. A number of findings suggest that, at least in part, this metabolism defect might be secondary to astrocyte dysfunction.

## Effects of Aging on Astrocytes

Aging affects many functional brain characteristics regulated by astrocytes, e.g., synaptic plasticity, metabolic balance, and BBB permeability. Increased expression of GFAP and vimentin has been the most common change observed in astrocytes with aging (132, 133). Interestingly, TGFβ1 signaling increases in the aging brain and can not only inhibit astrocyte proliferation but also stimulate GFAP expression. Furthermore, TGFβ1 is considered one of the main inducers of the senescence-associated secretory phenotype (SASP) observed in other cell populations (134, 135). Notably, senescent astrocytes can repress their capacity to support neuronal survival and neurite outgrowth, causing changes resembling those observed in the SASP, namely (1) increased expression of GFAP and vimentin filaments (132, 133); (2) accumulation of membrane-bound inclusion material in cytoplasm that appears to be lipofucsin, and ultraestructural changes in nuclei (136); and (3) increased expression of pro-inflammatory cytokines such as IL-6, TNF-α, IL-1β, and prostaglandins, which can enhance BBB permeability (134, 137). This age-related dysfunction can alter Ca^++^ homeostasis, and induce purinergic signaling in the gliovascular interface (138). In addition, astrocytes can release high-mobility group box-1 (HMGB1) protein, which promotes secretion of a specific subset of inflammatory factors, such as MMP-9, cyclo-oxygenase-2, and other chemokines facilitating monocyte infiltration (139). Indeed, during EAE progression, total and extracellular HMGB1 in the spinal cord is increased, and more positive astrocytes, neurons, and microglia are observed. Local block of CNS HMGB1 significantly attenuates EAE severity, suggesting HMGB1 expression in the spinal cord is associated with EAE progression (140).

Overall, inflammatory mediators appear to generate a vicious age-dependent cycle, where cellular senescence induces a low level of chronic inflammation, enhancing acute pathological conditions, and aggravating age-related neurodegenerative processes. This occurs through triggering of NO-induced pathways, and ROS-mediated dysfunction in mitochondria and endoplasmic reticulum.

## Concluding Remarks

**Figure 1:**
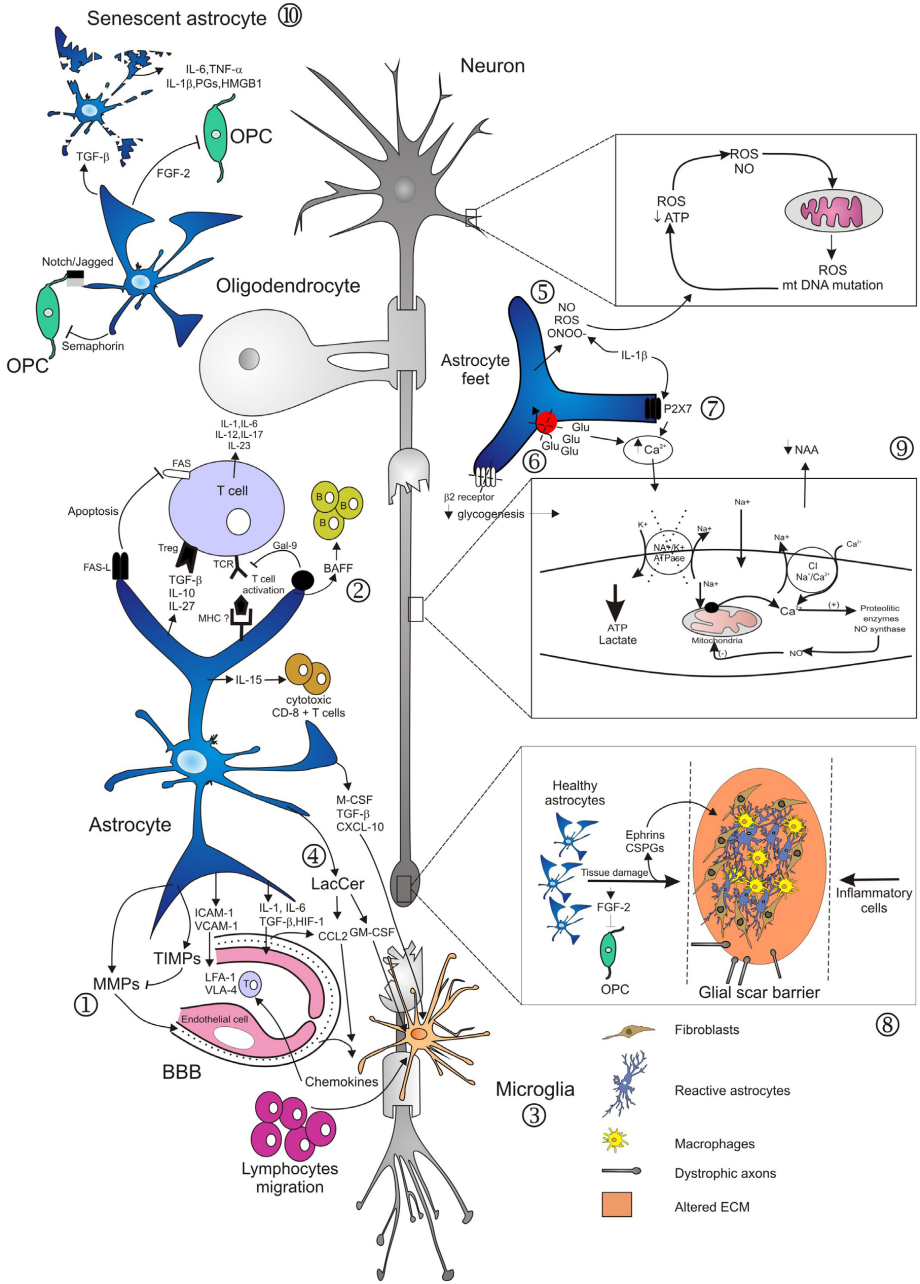
**Main mechanisms involved in neurodegeneration driven by astrocytes**. Several studies have demonstrated diverse roles of astrocytes in lesion development during the course of MS. Activation of astrocytes and loss of end-feet around small vessels are early events in lesion development, associated to loss of BBB function and consequently to CNS inflammation (1). Astrocytes mediate innate immune responses through several mechanisms. They modulate cell entry into the CNS by regulating adhesion molecule expression profiles, particularly of VCAM-1 and ICAM-1 (1). Astrocytes may also affect the number and phenotype of T cells in the CNS, committing T cells to a pro-inflammatory or regulatory phenotype. By contrast, astrocytes may also terminate T cell response, either by induction of apoptosis, or induction of Galectin-9. Furthermore, production of IL-15 or of BAFF drives immune responses mediated by cytotoxic CD8^+^ T cells or by B cells (2). Activated astrocytes secrete different chemokines, which attract both peripheral immune cells and microglia to MS lesions (2, 3). In the EAE model, astrocytes produce LacCer during the chronic phase, leading to induction of GM-CSF and CCL2 genes, and to subsequent microglial activation and monocyte infiltration (4). In areas of myelin breakdown, it has been documented that astrocytes secrete compounds with toxic effects for neurons, axons, and oligodendrocytes (5), reduce glutamate uptake by astrocyte transporters (6), and increase expression of purinergic receptors (7). These factors contribute to loss of glutamate buffering capacity mediated by astrocytes, mitochondrial dysfunction, energy deficiency, accumulation of intra-axonal Ca^2+^, and subsequent activation of proteolitic enzymes (9). Astrocytes respond to injuries by forming a glial scar that inhibits remyelination and axonal regeneration. These effects are mediated through secretion of fibroblast growth factor-2 (FGF-2) and of inhibitory extracellular matrix (ECM) molecules, such as condroitin sulfate proteoglycans (CSPGs) and Ephrins (8). Old age adversely affects astrocyte viability and self-renewal capacity, resulting in the generation of senescent and/or dysfunctional cells, evidenced in the form of cell fragmentation (10). Senescent astrocytes appear to be in a state of chronic activation, associated with pro-inflammatory cytokine and prostaglandins secretion.

Astrocytes are a diverse cell population, differing across the CNS in their morphology, physiology, and function. In recent years, growing evidence indicates that astrocytes are more than simple bystander cells providing an optimal physical and metabolic environment for neuronal activity. In MS lesions, they exert active, dual, and paradoxical roles during disease development (39). Some experimental data implicate astrocytes as actual mediators of inflammation, as observed in sites of injury, ultimately limiting neuronal repair and remyelination (Figure 1). By contrast, other evidence suggests astrocytes curtail detrimental effects of pro-inflammatory factors, thus providing support and protection for oligodendrocytes and neurons. This dichotomy in astrocyte effects makes designing new therapeutic strategies targeting astrocytes a challenging endeavor. In this context, a better definition of astroglial subtypes based on their molecular, functional, and structural properties, should greatly promote our understanding of their specific roles in MS pathophysiology, and consequently lead to the development of novel cell-targeting therapies for the disease.

## Author Contributions

JC conceived the manuscript, outlined the subject of the review, searched for, analyzed and interpreted the literature, wrote the manuscript, edited and revised it for important intellectual content, gave final approval of the version for publication, and accepts full accountability for all aspects of the work. MF edited and revised the manuscript for important intellectual content, gave final approval of the version for publication, and accepts accountability for all aspects of the work.

## Conflict of Interest Statement

Jorge Correale is a board member of Merck-Serono Argentina, Novartis Argentina, Genzyme LATAM, Genzyem global, Biogen-Idec LATAM, and Merck-Serono LATAM. Dr. Jorge Correale has received reimbursement for developing educational presentations for Merck-Serono Argentina, Merck-Serono LATAM, Biogen-Idec Argentina, Genzyme Argentina, Novartis Argentian, Novartis LATAM, and TEVA Argentina as well as professional travel/accommodations stipends. Mauricio F. Farez has received professional travel/accommodations stipends from Merck-Serono Argentina and Novartis Argentina.
